# User - provider perspectives to overcome the challenges of TB/HIV service integration at Mulago National Referral Hospital _ Kampala

**DOI:** 10.4314/ahs.v21i1.32

**Published:** 2021-03

**Authors:** Jane Namakula Katende, Kizito Omona

**Affiliations:** 1 Bachelor of Social work and Social Development and Master of Public Health (MPH). Faulty of Health sciences, Uganda martyrs University. janekatende9@gmail.com, Tel; +256772302503; 2 Uganda Martyrs University, Faculty of Health Sciences. kizitoomona@gmail.com, Tel; +256706464873

**Keywords:** User - provider perspectives, TB/HIV service integration, Mulago National Referral Hospital

## Abstract

**Background:**

Tuberculosis and Human Immunodeficiency Virus epidemics in sub-Saharan Africa have been closely related and persistent, proving a considerable burden for healthcare provision. This has complicated utilization of services, with noted opinions on the integration of these services from both users and providers of the services.

**Objectives:**

To establish the users and the provider's perspectives in overcoming the challenges of TB/HIV services integration at Mulago National Referral Hospital.

**Methods:**

Descriptive cross-sectional design, with predominantly qualitative methods was used. Qualitative aspect adopted phenomenological design. Participants were randomly selected for FGDs and Key informants. An observation checklist collected quantitative data from the patients to measure level of services integration.

**Findings:**

Level of service integration of TB/HIV services was at 68% (below the acceptable 100% level). Opinions from the users pointed to; increasing number of work-days for TB/HIV service provision, strengthening sensitisation and health education and integrating other services like reproductive health services, among others. Health care providers opinions pointed to increasing trainings for health workers, increasing staffing and need for more support from Ministry of Health.

**Conclusion:**

Opinions from both users and providers were similar. These ranged from increasing awareness to the users and healthcare providers about the integration of services.

## Introduction

### Background to the Study

The World Health Organization (WHO) defines integrated healthcare as ‘the organization and management of health services so that people get the care they need, when they need it, in ways that are user-friendly, achieve the desired results and provide value for money 1. The Tuberculosis (TB) and Human Immunodeficiency Virus (HIV) pandemics in sub-Saharan Africa have been closely related and particularly persistent, proving a considerable burden for healthcare provision and hence complicated the utilization of services, with noted opinions on the integration of these services 2. The impact of HIV on TB, and the implications for TB and HIV control, has been acknowledged as a public health challenge in Uganda, as is the case in many other sub-Saharan African countries 3.

In the early years, TB and HIV services were disconnected, which meant an increase in the cost of care for patients, as well as other added inconveniences, as numerous visits were required to access the required care. There were higher losses to follow-up and case fatalities, as well as delays in Anti-retroviral Therapy (ART) initiation^4^. Although TB programme indicators like case notification, default rates and case evaluation has been progressively improving under the existing TB control interventions, death rates were high and treatment success remained below the global target of 85% and this has been probably due to lack of good integration of the services 5.

In the year 2015, there were an estimated 10.4 million new TB cases worldwide and people living with HIV accounted for 1.2 million (11%) of all the new TB cases^6^. In 2014, the World Health Organization (WHO) reported 83% incident TB cases worldwide out of which one third of these new TB cases originated from the African continent with high burden countries (HBCs)^7^. A similar trend was documented in the 2016. WHO global TB report which showed that the proportion of TB cases living with HIV was highest in the WHO African Region (31%) and exceeded 50% in parts of southern Africa^6^,^7^.

In 2007, South Africa, with 0•7% of the world's population, had 17% of the global burden of HIV infection, and one of the world's worst tuberculosis epidemics, compounded by rising drug resistance and HIV co-infection. Until recently, the South African Government's response to these diseases has been marked by denial, lack of political will and poor implementation of integration policies and programs 8.

Uganda is among the 22 countries considered by WHO as TB high-burden countries in the world 9. To decrease the joint burden of HIV and TB disease, the World Health Organization (WHO) formulated a strategic framework for collaborative TB/HIV activities. While patients receive these services, they are faced with several challenges that need to be sorted out so that recommendations can be drawn for effective utilization of these services 6.

The integrated service delivery models in the views of the health care provider should focus on the one patient with two life-threatening diseases (TB/HIV) who often has to navigate complex health systems to secure access to care and treatment. However, in many settings with the greatest impact of TB and HIV, care integrated within one facility has always been impeded due to many shortfalls like lack of training on the side of staffs to handle both cases of disease, separate programme management and geographical separation of services^10^. There has been persistent differing views of healthcare providers and users in few quantitative surveys that assessed provider-user perspectives and interactions on quality of integrated healthcare programs including TB/HIV services 1. This provides evidence that pins the fact that there are numerous challenges impeding the TB/HIV service delivery integration across the board and a study like this will, most likely, bring out those issues from both the views of users and the providers to help in overcoming these challenges.

This study aimed to establish the users and the provider's perspectives in overcoming the challenges of TB/HIV services integration at Mulago national referral Hospital so that these services are made accessible and affordable to patients.

### Objectives of the Study

Broad Objective: To establish the users and the provider's perspectives in overcoming the challenges of TB/HIV services integration at Mulago national referral Hospital so that these services are made accessible and affordable to patients.

**Specific objectives: The specific objectives were;**

1. To determine the user's level of TB/HIV services integration at Mulago National Referral Hospital _ Uganda
2. To examine the User's opinions in overcoming the challenges of TB/HIV services integration at Mulago National Referral Hospital _ Uganda
3. To find out the provider's views in overcoming the challenges of TB/HIV services integration at Mulago National Referral Hospital _ Uganda
4. To establish the challenges faced by users in accessing TB/HIV services at Mulago National Referral Hospital _ Uganda, TB/HIV ward

## Methods

The study was a descriptive cross-sectional study done on TB/HIV co-infected patients attending TB/HIV clinic. It was a mixed methods study but predominantly qualitative in nature. Very little/few aspects of this was quantitative methods. The study targeted adult HIV/TB patients on ART and receiving both ART and TB services from Mulago National referral Hospital TB/HIV clinic. Health care workers working in the TB and HIV clinic gave views on TB/HIV integrated services including doctors, Nurses, ART counsellors, pharmacy technicians and laboratory technologists. Sample size was calculated using Kish Leslie Single proportion model and generation of 139 participants was made for the quantitative part. However, three (3) focus group discussions and 10 key informant interviews generated the qualitative data required.

Simple random sampling was used to select the study participants because there was an equal chance (probability) of selecting each TB/HIV co-infected patient from the population being studied. An observation checklist collected quantitative data. A focus group discussion guide and an interview guide with open-ended questions collected primary data from the users and providers for qualitative information respectively.

Permission to carry out the study was obtained from the Department of Health sciences of Uganda martyrs University, Mulago National Referral Hospital Research and Ethics Committee and the head of Mulago TB unit in that respective order. Voluntary informed consents of the respondents were sought through a guided written informed consent (in English or Luganda - the common local language used). Explanation of the objectives and benefits of the study was made known to the respondents. The respondents then signed or put a thumbprint on the consent form after voluntarily accepting to participate in the study.

## Findings

### Socio-demographic characteristics

The study was conducted on 139 participants. Majority 89(64%) of the participants were male compared to their female counterparts 50(36%). See details in table 1 below;

**Table 1:** Socio-demographic Characteristics of the Respondents

Variables	Frequency(n=139)	Percent (100%)
**Gender**		
▪ Male ▪ Female	89 50	64 36

**Age of respondents in years**		
▪ 18–30years ▪ 31–40 ▪ 41–50	40 55 44	29 40 31

**Level of education**		
▪ None ▪ Primary ▪ Secondary ▪ Tertiary	5 59 64 11	4 42 46 8

**Marital status**		
▪ Single ▪ Married ▪ Divorced/separated ▪ Widowed	30 65 35 9	22 47 25 6

**Religion**		
▪ Catholics ▪ Protestant ▪ Born again ▪ 7^th^ Day Adventist ▪ Moslem	67 40 14 3 15	48 29 10 2 11

### User's level of TB/HIV services integration

User's level of TB/HIV services integration of all the services in the clinic stood out at 68% for patients who saw the same provider, got both TB/HIV drugs and those who got the same appointment for TB/HIV services.

### User's opinions in overcoming the challenges of TB/HIV services integration

A number of themes were generated and findings reported as per the respective theme below;

#### Theme 1a: Rationale of TB/HIV Services Integration

FGD's pointed out that TB/HIV services integration help to improve good adherence to the treatment as opposed to the way it was being done on the different days where some patients miss their doses because it was being done on different days. This was expressed as below;

“*[…] At one time I had travelled to the village for the burial of one of my relatives but had only checked my stock of ARV's, only to check during my dosing time, I realised that the TB medicines were done and this caused me to miss my appointment and the doses until I came back to Kampala*” FGD 1

The integration of TB/HIV services can help clients to save time and transport. Patients always found it difficult to ask for permission to visit the hospital every after two weeks, yet if drugs were given to them at once, it may be that this would reduce to at least one-visit per month. One client stressed this in the following words; “*When we are treated and we get better we go back to work and sometimes too far and distant places. The challenge is when we are made to come at different times to pick drugs for both TB and HIV […]*” FGD 2.

#### Theme 2a: Opinions on the staffing structure of TBH ward

Participants in all the FGD's thought that there was need for more staffing at the clinic. Participants gave an example of the pharmacy window, where some of them said that they would only see one person serving them since they started getting drugs for TB. They said the reverse is true for those cliens getting HIV drugs.

However, other participants, from all the FGDs thought that the staffing structure was enough in general, only if the health workers were doing the right things at the right time

“*The staffing levels at this clinic are fare compared to other government facilities/clinics we go to. The only issue that affects us hear is the way these staff organise to do things. Some patients get lost with in the clinic, they start late and all these disorganise the patient flow resulting into the whole clinic being messed up not that staffs are few but the process or system issue what one can call the client flow […]*” FGD 3.

#### Theme 3a: Opinions to improve TB/HIV services integration

Participants expressed the need for the ward management to sit down and see a way of increasing the number of days they see patients who are co-infected with both TB and HIV. One day is not enough and if this can be increased to two or more days, the better.

One patient said, “*For us as patients, we shall be grateful for this innovation*” -FGD 2

And, another patient said;

“*[…]A Friday is not the best day for all of us, I quite often miss my days because I attend to clients at the office alone on Friday at my station up to 1Pm. When I come, sometimes I am late and everyone has labelled me as a late comer but this is not because of my own making. I request for a revision into this arrangement*”-FGD2.

### Provider's opinions in overcoming the challenges of TB/HIV Services Integration

A number of themes were generated and findings reported as per the respective theme below;

#### Theme 1b: Level of TB/HIV Services Integration at Mulago Ward 5 & 6

The level of TB/HIV services integration was fair and the basis for this was due to the fact that services are offered on Fridays only and this affects those patients who are diagnosed on other days of the week since they cannot access ART services. This was said below;

“*[…] I can say that the level of TB/HIV services integration at this clinic is fairly sub optimal and there is need to intensify these services so that patients can access treatment at the same point by the same service provider and on the same appointment*” -Key Informant 6.

#### Theme 2b: Staffing levels at the Mulago TBH Ward

Staffing levels of the ward was found to be very low. Most health care providers narrated how the Friday clinic makes them over work and the number of patients is usually too many to be handled by the few staff available. One of the KI's noted the following;

“*The staffing level at the TB/HIV clinic is still wanting since the few staffs who are available have to make sure that all the patients get their care on time […]*” Key Informant 2.

The situation worsened on Fridays when the TB/HIV clinic is usually heavy and the ratio even went up to about 1:50, that is to say, 1 Doctor for every 50 patients. The health care providers further narrated that the low patient - health care provider ratio contributes to the long waiting time that can be vividly seen by everyone who comes to the clinic.

#### Theme 3b: Trainings in TB/HIV Services Integration

Sixty percent (6 out of 10) of the key informants had trainings in the TB/HIV services integration while the remaining did not have any training. However, it was noted that the implementing partner handled even those staff who had ever got trainings, these trainings, which is Makerere University Joint AIDS program (MJAP). These also expressed the need for more trainings in this field since these trainings had taken place many years ago.

#### Theme 4b: Suggested Measures to Improve the Integration of TB/HIV Services

Majority of the Key Informants (KIs) interviewed (6 out of 10) overwhelmingly supported the idea of recruiting more staff at the TB/HIV clinic to handle the large numbers of clients who report for service at the clinic. This in, a way, can ease the integration of both services, such that the staff and management of Mulago ward 5 and 6 do not have to depend on the implementing partner to support the TB/HIV services.

All the KIs interviewed agreed that there is need for more trainings of the staffs who support the implementation of TB/HIV services. Sometimes, failure of implementation of these services is because of lack of knowledge of some health care workers and if regularly trained and updated in its implementation, they would see better outcome. KI, James [not real name], stressed this point as follows;

[…] Sometimes I don't understand what these MJAP people ‘the implementing partner’ always insist on, that integration, integration…, the fact is that we have always treated these people the way we do it and they cure from TB then after continue with ARV's -Key Informant 1

### Challenges faced by patients in accessing TB/HIV integrated services at Mulago TB/HIV Ward

Lack of inappropriate well-coordinated TB/HIV services was mentioned by all the members in the FGDs as one of the biggest challenges affecting its implementation. Most of the members confessed to the discussion that when they report early, the staffs come late and even then, when they (staff) report, they start moving around without any concern of how delayed they had been. Participant 2, FGD 3 had the following to say in her own words;

“*[…] even after being worked on by the doctors, you can see most patients lost in the compound of this ward because sometimes the window is still closed yet they have been sent to pick medicines. So, patients tend to think that maybe they have been sent to another place*” -FGD 3

Lack of treatment for the other opportunistic infections was another huge challenge. All the participants agreed that, it is indeed, a huge challenge for someone to go to the hospital for a service and get treated, when you have any other health related illness. A participant in the FDG 2 had this to say;

“*[…] Sometimes when you negotiate with the health care provider, he can write for you these drugs but they are too expensive in the pharmacies around Kampala, yet you are struggling with buying food and other needs of life at home. If possible the government should also think of making drugs for other infections like malaria available for us to have free access just like it is for TB and HIV drugs*” FGD 2.

## Discussion

Findings from this study observed that there exists some level of TB/HIV services integration. It was, however, noted that the biggest challenge of this service integration was in patients getting the same clinic appointment from the service providers, among others. Similar findings were reported by a study^11^ in Malawi on integrated tuberculosis and HIV care which showed that ART acceptance among TB patients was low due to transport costs from home to the centralized hospital ART site where there was no TB integration.

Most participants in the FGD's gave their opinion on the low staffing of health workers as one of the challenges faced in accessing the TB/HIV services integration. This phenomenon is the same across all the healthcare system. This is similar to the findings of the study done in Uganda^12^ to evaluate the health care providers' and Clients' perspectives on the quality of tuberculosis services in health care centers. The study found out that understaffing was one of the factors affecting the delivery thee services.

The processes and the organization of the services at the clinic was found wanting and needed improvement according to the opinions of the participant's. Health workers' attitude of late coming, the way the staff organize to do things and at what time were among relevant issues expressed by participants that needed to be improved. This is in agreement with the study done in Peru that identified poor coordination of services at the programmatic level as one of the challenges facing TB/HIV integration of services^13^.

Harmonization of appointments for TB/HIV clinic was a way forward noted by users and providers to help in easing client's access to these services alongside with working on improving the staffing gap. In fact, some patients can think that the healthcare provider has decided to offer it that way and it ends up compromising the quality of service delivery and treatment outcomes due to poor adherence. These findings are in agreement with those of another author^14^, who found out that patients had faced substantial time costs associated with care seeking, primarily due to frequent travels to clinic visits and this too influenced HIV/TB treatment uptake, adherence and retention.

## Study limitation

The study had two major limitations; (1) Being predominantly qualitative in nature, the findings are not generalizable (2) Being cross-sectional, the trends of the findings cannot be traced.

## Implication of the study

Given the results of the study, there is need to revamp, sustain and consolidate efforts toward TB/HIV service integration in the hospital. This is because the challenges were not out of proportion.

## Conclusion

The level of TB/HIV services integration at the Mulago hospital ward 5 and 6 was fair, although it is offered with many challenges faced by the users of this service. User's opinions were almost similar to those of providers and these ranged from increasing awareness to the users and healthcare providers about the integration of the service in order to improve the mechanisms through which this service can best be implemented. Engagement of all the stakeholders is key in this improvement.

## References

[1] Ameh S, Klipstein-Grobusch K, D'ambruoso L, Kahn K, Tollman SM, Gómez-Olivé FX (2017). Quality of integrated chronic disease care in rural South Africa: user and provider perspectives. Health Policy and Planning.

[2] Naidoo K, Gengiah S, Yende-Zuma N, Padayatchi N, Barker P, Nunn A (2017). Addressing challenges in scaling up TB and HIV treatment integration in rural primary healthcare clinics in South Africa (SUTHI): a cluster randomized controlled trial protocol. Implementation Science.

[3] Okot-Chono R, Mugisha F, Adatu F, Madraa E, Dlodlo R, Fujiwara P (2009). Health system barriers affecting the implementation of collaborative TB-HIV services in Uganda. The International Journal of Tuberculosis and Lung Disease.

[4] Ansa GA, Walley JD, Siddiqi K, Wei X (2012). Assessing the impact of TB/HIV services integration on TB treatment outcomes and their relevance in TB/HIV monitoring in Ghana. Infectious Diseases of Poverty.

[5] Lönnroth K, Castro KG, Chakaya JM, Chauhan LS, Floyd K, Glaziou P (2010). Tuberculosis control and elimination 2010–50: cure, care, and social development. The Lancet.

[6] World Health Organisation (2016). Global tuberculosis report 2016.

[7] World health Organisation (2015). WHO Global report.

[8] Zhuwau T, Ndlovu N, Naidu V, Ngwenya T, Mkhize S (2017). community-led integrated Service delivery ‘War rooms’ and the fight against hiV, aidS and tB in Kwa-Zulu-natal province, South africa. Sizonqoba!. Outliving AIDS in Southern Africa.

[9] Hermans SM, Castelnuovo B, Katabira C, Mbidde P, Lange JM, Hoepelman AI (2012). Integration of HIV and TB services results in improved TB treatment outcomes and earlier, prioritized ART initiation in a large urban HIV clinic in Uganda. Journal of Acquired Immune Deficiency Syndromes (1999).

[10] Legido-Quigley H, Montgomery CM, Khan P, Atun R, Fakoya A, Getahun H (2013). Integrating tuberculosis and HIV services in low-and middle-income countries: a systematic review. Tropical Medicine & International Health.

[11] Phiri S, Khan P, Grant A, Gareta D, Tweya H, Kalulu M (2011). Integrated tuberculosis and HIV care in a resource-limited setting: experience from the Martin Preuss centre, Malawi. Tropical Medicine & International Health.

[12] Bulage L, Sekandi J, Kigenyi O, Mupere E (2014). The quality of tuberculosis services in health care centres in a rural district in Uganda: the providers' and clients' perspective. Tuberculosis Research and Treatment.

[13] Garcia-Fernandez L, Benites C, Huaman B (2017). Access barriers to comprehensive care for people affected by tuberculosis and human immunodeficiency virus coinfection in Peru, 2010–2015. Rev Panam Salud Publica.

[14] Chimbindi NZ (2017). HIV and TB care and treatment: patient utilization and provider perspectives in rural KwaZulu-Natal.

